# Improving care of migrants is key for viral hepatitis elimination in Europe

**DOI:** 10.2471/BLT.20.260919

**Published:** 2021-01-21

**Authors:** Jin Un Kim, Patrick Ingiliz, Yusuke Shimakawa, Maud Lemoine

**Affiliations:** a Hepatology Section, Division of Digestive Diseases, Department of Metabolism, Digestion and Reproduction, 10th Floor QEQM Wing, St Mary’s Hospital Campus, Imperial College London, South Wharf Street, London W2 1NY, England.; b Department of Gastroenterology and Hepatology, Charité University Medical Center, Berlin, Germany.; c Unité d'Épidémiologie des Maladies Émergentes, Institut Pasteur, Paris, France.

## Abstract

By 2040, deaths from chronic viral hepatitis worldwide are projected to exceed those from human immunodeficiency virus infection, tuberculosis and malaria combined. The burden of this disease is predominantly carried by low-resource countries in Africa and Asia. In resource-rich countries, the epidemiological spread of viral hepatitis is partially driven by migrant movements from areas of high endemicity. In the last decade, Member States of the European Union and the European Economic Area have experienced an unprecedented influx of migrants, which has resulted in the polarization of political views about migration. In addition, the coronavirus disease 2019 pandemic has worsened the economic and health conditions of migrants and contributed to hostility to ensuring their health rights. Moreover, the implementation of hostile laws in some host nations has increased the vulnerability of marginalized migrant subgroups, such as asylum seekers and undocumented individuals. These developments have complicated the historical challenge of identifying high-risk migrant groups for screening and treatment. However, if European countries can apply the simplified assessment tools and diagnostic tests for viral hepatitis that have been used for decentralized screening and monitoring in resource-poor countries, the uptake of care by migrants could be dramatically increased. Given the global calls for the elimination of viral hepatitis, European nations should recognize the importance of treating this vulnerable migrant population. Political and health strategies need to be adapted to meet this challenge and help eliminate viral hepatitis globally.

## Résumé

D'ici 2040, les décès causés par l'hépatite virale chronique dans le monde devraient dépasser ceux dus à trois grandes maladies réunies: l'infection au virus de l'immunodéficience humaine, la tuberculose et la malaria. Le fardeau que représente cette affection repose surtout sur les pays disposant de ressources limitées en Afrique et en Asie. Dans les pays riches en ressources, la propagation épidémiologique de l'hépatite virale est en partie liée aux mouvements migratoires depuis les zones à endémicité élevée. Au cours de la dernière décennie, les États membres de l'Union européenne et l'Espace économique européen ont connu un afflux de migrants sans précédent qui a polarisé les opinions politiques concernant la migration. En outre, la pandémie de maladie à coronavirus 2019 a aggravé la situation économique et sanitaire des migrants, contribuant à l'animosité ambiante à l'égard du respect de leurs droits en matière de santé. L'adoption de lois hostiles dans certains pays d'accueil a également accru la vulnérabilité des sous-groupes de migrants marginalisés, tels que les demandeurs d'asile et les sans-papiers. Des conditions qui compliquent la tâche d'identification des groupes de migrants à haut risque pour le dépistage et le traitement. Néanmoins, si les pays européens pouvaient appliquer les outils d'évaluation simplifiés et les tests de diagnostic de l'hépatite virale, qui ont été employés pour la surveillance et le dépistage décentralisé dans les pays disposant de ressources limitées, la prise en charge des migrants pourrait nettement s'améliorer. Compte tenu des nombreux appels internationaux à éliminer l'hépatite virale, les nations européennes devraient reconnaître l'importance de soigner ces populations de migrants vulnérables. Les stratégies politiques et sanitaires doivent être adaptées afin de relever ce défi et de contribuer à éradiquer l'hépatite virale dans le monde.

## Resumen

Para 2040, se prevé que las muertes por hepatitis vírica crónica en todo el mundo superen a las causadas por la infección del virus de la inmunodeficiencia humana, la tuberculosis y la malaria juntas. La carga de esta enfermedad recae sobre todo en los países con recursos limitados de África y Asia. En los países ricos en recursos, la propagación epidemiológica de las hepatitis víricas se debe en parte a los movimientos migratorios desde las zonas altamente endémicas. En la última década, los Estados miembros de la Unión Europea y del Espacio Económico Europeo han experimentado una afluencia de inmigrantes sin precedentes, lo que ha polarizado las opiniones políticas sobre la inmigración. Además, la pandemia de la enfermedad del coronavirus de 2019 ha empeorado las condiciones económicas y sanitarias de los inmigrantes y ha contribuido a la hostilidad para garantizar sus derechos sanitarios. Además, la aplicación de leyes hostiles en algunas naciones de acogida ha aumentado la vulnerabilidad de subgrupos de inmigrantes marginados, como los solicitantes de asilo y los indocumentados. Estos acontecimientos han complicado el reto histórico de identificar a los grupos de inmigrantes de alto riesgo para su detección y tratamiento. Sin embargo, si los países europeos pueden aplicar las herramientas de evaluación y las pruebas de diagnóstico simplificadas para la hepatitis vírica que se han utilizado para el cribado y el seguimiento descentralizados en los países con pocos recursos, la aceptación de la atención por parte de los inmigrantes podría aumentar drásticamente. Dados los llamamientos mundiales para la eliminación de la hepatitis vírica, las naciones europeas deberían reconocer la importancia de tratar a esta población inmigrante vulnerable. Es necesario adaptar las estrategias políticas y sanitarias para hacer frente a este reto y ayudar a eliminar la hepatitis vírica a nivel mundial.

## ملخص

بحلول عام 2040، من المتوقع لحالات الوفاة الناجمة عن التهاب الكبد الفيروسي المزمن في جميع أنحاء العالم، أن تتجاوز الوفيات الناجمة عن عدوى فيروس نقص المناعة البشرية، والسل، والملاريا مجتمعة. يتحمل عبء هذا المرض في المقام الأول البلدان منخفضة الموارد في إفريقيا وآسيا. أما في البلدان الغنية بالموارد، فيكون الانتشار الوبائي لالتهاب الكبد الفيروسي مدفوعًا جزئيًا بتحركات المهاجرين من مناطق ذات استشراء مرتفع للوباء. شهدت الدول الأعضاء في الاتحاد الأوروبي والمنطقة الاقتصادية الأوروبية في العقد الماضي تدفقاً غير مسبوق للمهاجرين، وهو مما أدى إلى استقطاب للآراء السياسية حول الهجرة. بالإضافة إلى ذلك، أدت جائحة فيروس كورونا 2019 إلى تفاقم الظروف الاقتصادية والصحية للمهاجرين، وساهمت في العداء تجاه ضمان حقوقهم الصحية. وبالإضافة لذلك، أدى تنفيذ القوانين المعادية في بعض الدول المضيفة إلى زيادة ضعف المجموعات الفرعية المهمشة من المهاجرين، مثل طالبي اللجوء والأفراد غير المسجلين. أدت هذه التطورات إلى تعقيد التحدي التاريخي الخاص بتحديد مجموعات المهاجرين عالية الخطورة، ليتم فحصهم وعلاجهم. وبالرغم مع ذلك، فإنه إذا كانت الدول الأوروبية تستطيع تطبيق أدوات التقييم المبسطة والاختبارات التشخيصية لالتهاب الكبد الفيروسي، والتي تم استخدامها للفحص والمراقبة اللامركزية في البلدان ذات الموارد الفقيرة، فإنه يمكن زيادة حصول المهاجرين على الرعاية بشكل كبير. مع وضع الدعوات العالمية للقضاء على التهاب الكبد الفيروسي في الاعتبار، يجب على الدول الأوروبية أن تدرك أهمية علاج هؤلاء السكان المهاجرين المعرضين للخطر. تحتاج الاستراتيجيات السياسية والصحية للتكيف حتى تواجه هذا التحدي وتساعد في القضاء على التهاب الكبد الفيروسي على مستوى العالم.

## 摘要

到 2040 年，全球范围内因慢性病毒性肝炎导致的死亡人数预计将超过人类免疫缺陷病毒感染、结核病与疟疾导致的总死亡人数。在治疗这种疾病方面，非洲和亚洲那些资源匮乏的国家负担最重。在资源丰富的国家中，流行病学调查结果表明，病毒性肝炎的传播部分是因来自高流行地区的移民迁移造成的。在过去十年中，欧盟和欧洲经济区成员国经历了前所未有的移民潮，这导致了关于移民的政治观点出现两极化。此外，新型冠状病毒肺炎疫情使移民的经济状况和健康状况出现恶化并助长了敌对情绪，导致难以确保他们的保健权。此外，在一些东道国中实施怀有敌意的法律使边缘化移民小群体（例如寻求庇护者和无证人员）增加了患病的可能性。这些事态发展使我们在确定需要筛查和治疗的高风险移民群体方面所面临的历史挑战变得日益复杂。然而，如果欧洲国家可以采用在资源匮乏国家中用于分散筛查和监测病毒性肝炎的简化评估工具和诊断测试，则可以大幅提高移民的医疗保健覆盖率。鉴于全球呼吁消除病毒性肝炎，欧洲国家应该认识到治疗此类极易受感染的移民群体的重要性。目前需要调整政治和卫生策略来应对这一挑战并帮助在全球范围内消除病毒性肝炎。

## Резюме

Согласно прогнозам, к 2040 году смертность от хронического вирусного гепатита во всем мире превысит смертность от вируса иммунодефицита человека, туберкулеза и малярии, вместе взятых. Бремя этой болезни преимущественно несут страны Африки и Азии с ограниченными ресурсами. В богатых ресурсами странах эпидемиологическое распространение вирусного гепатита частично обусловлено перемещениями мигрантов из районов с высокой эндемичностью. За последнее десятилетие государства-члены Европейского союза и Европейской экономической зоны испытали беспрецедентный приток мигрантов, который привел к поляризации политических взглядов на миграцию. Кроме того, пандемия коронавирусного заболевания 2019 года ухудшила экономические условия и состояние здоровья мигрантов и поспособствовала враждебному отношению к обеспечению их прав на здоровье. Более того, реализация враждебных законов в некоторых принимающих странах повысила уязвимость отчужденных подгрупп мигрантов, таких как лица, ищущие убежища, и лица без документов. Эти события усложнили уже существующую задачу по выявлению групп мигрантов высокого риска для скринингового обследования и лечения. Однако, если европейские страны смогут применить упрощенные инструменты оценки и диагностические тесты на вирусный гепатит, которые использовались для децентрализованного скринингового обследования и мониторинга в странах с ограниченными ресурсами, обращение за медицинской помощью со стороны мигрантов может резко возрасти. С учетом глобальных призывов к ликвидации вирусного гепатита европейские страны должны признать важность лечения этой уязвимой группы мигрантов. Для решения этой проблемы и помощи в ликвидации вирусного гепатита во всем мире необходимо адаптировать политические стратегии и стратегии в области здравоохранения.

## Introduction

Viral hepatitis is a global health concern and causes around 1.3 million deaths each year,^1^ mainly as a result of chronic liver disease and its complications. Around 96% of viral hepatitis deaths in 2015 were attributable to chronic hepatitis B virus (HBV) and hepatitis C virus (HCV) infections, which affected 257 million and 71 million people, respectively.^1^^,^^2^ Worldwide, the disease prevalence is spread disproportionately: 20 countries, the majority being resource-constrained countries in Asia and sub-Saharan Africa, carry 75% of the global hepatitis burden.^3^

Despite the high health burden of viral hepatitis, international efforts to tackle the condition have only recently begun. In 2016, the World Health Organization (WHO) called for an ambitious strategy to eliminate viral hepatitis globally – the initial targets were a 90% reduction in the number of new infections by 2030 compared to 2015 and a reduction of 65% in the number of deaths.^2^ Subsequently, major progress has been made on an international scale: (i) WHO’s global health sector strategy on viral hepatitis was developed;^2^ (ii) mother-to-child transmission of HBV was reduced in WHO’s Western Pacific Region;^4^ (iii) HCV elimination plans were established in Egypt and Georgia;^5^ and (iv) numerous global hepatitis elimination initiatives were launched.^3^^,^^6^

These initiatives and recent research have tended to focus on identifying effective diagnostic and therapeutic modalities and on improving local cascades of care.^3^ However, specific population groups vulnerable to infection and marginalized by local health-care systems (e.g. undocumented migrants, people who inject drugs and prisoners) have been excluded from these developments and have been neglected by management strategies for viral hepatitis.^3^ In particular, migrants from endemic areas have long been recognized as forming a high-risk population for the disease and its complications.^7^^,^^8^ Despite their vulnerability, certain migrant subgroups, including undocumented migrants and asylum seekers, have historically been difficult to target through existing surveillance and care pathways because of social, political and cultural barriers.^9^ Moreover, the extent to which migrants are integrated into national health and welfare systems differs between European countries. In general, undocumented migrants have almost no access to the formal health-care system outside of emergency care and, in addition, migrants are vulnerable to xenophobic discrimination, which can lead to social exclusion.^10^ In 2017, a health system survey by the Migration Integration Policy Index reported that health care for migrants had been not secured in most European countries.^11^

Migrants’ health and access to care have also been affected by the recent polarization of political views, which has resulted in many European countries becoming resistant to providing health care for migrant subgroups.^12^ In addition, the current antimigrant political climate in the United States of America is likely to increase immigration into Europe.^13^ To add to these already difficult circumstances, the ongoing coronavirus disease-2019 (COVID-19) pandemic has worsened both the immediate and long-term economic and health conditions of marginalized migrants.^14^

Globally only a few countries are on track to achieve viral hepatitis elimination goals and, without greater inclusion of vulnerable migrants in their health-care systems, many European countries are unlikely to achieve these goals within the next decade.^15^^,^^16^ The current negative political climate has created multiple barriers to providing health care for vulnerable individuals who may require screening, care and treatment for viral hepatitis; this can only hamper efforts to eliminate the disease. Rather than regarding migration as a health threat, European countries should see it as an opportunity to contribute to the global elimination of viral hepatitis.^17^

Here we discuss barriers to providing viral hepatitis care for vulnerable migrants and present suggestions for improvement.

## Political threat to health care 

The global migrant crisis in 2015 saw an unprecedented influx of first-time asylum seekers and undocumented migrants into the European Economic Area. Of the 4.3 million people who immigrated into European Union (EU) Member States during 2015, 1.3 million were first-time asylum seekers from conflict zones around Afghanistan, Iraq and the Syrian Arab Republic who applied for international protection.^18^^,^^19^ The number of asylum applications in 2015 was double the figure for the previous year and there were ongoing humanitarian concerns about refugees in Europe, such as those in the Moria refugee camp in Greece.^18^

Undoubtedly this was a critical moment when political views on the issue of migration divided European Member States, local communities and even families, ultimately contributing to the increased popularity of several far-right political parties. These polarized views were reflected, in part, by changes in health policy in several EU Member States following the migrant crisis. For example, five EU Member States (Croatia, Germany, Slovenia, Sweden and the United Kingdom of Great Britain and Northern Ireland) and Bosnia and Herzegovina (i) required health-care workers to report undocumented migrants attending clinical facilities to the immigration office; (ii) did not provide state-funded treatment for communicable diseases; and (iii) restricted access to services other than emergency care.^20^^,^^21^ Furthermore, sociocultural barriers deterred migrants (who may have had legal documentation entitling them to health care) from seeking care in the first place, including: (i) fear of deportation; (ii) financial concerns; (iii) a lack of information on their right to health care; and (iv) the likelihood they may migrate again and be lost to follow-up.^22^^,^^23^

In response to concerns that migrants were major contributors of communicable diseases within host nations, a recent *Lancet* Commission observed that migrants arriving in Europe often had better health than people who remained in their countries of origin – this was termed the “healthy migrant concept”.^24^ On the other hand, the *Lancet* Commission’s study recognized that some subgroups from specific geographical locations, who may have been vulnerable to infectious liver diseases in their home nations or during their journeys, were more likely to have or be at risk of contracting these diseases.^23^^,^^24^

Contrary to their sworn oaths of beneficence and non-maleficence, health-care workers have been unwillingly recruited into a political battle in which the provision of basic care has become conditional. The issue of viral hepatitis sits at a critical juncture in this complex situation because migrant flows tend to come from regions where viral hepatitis is highly endemic and because the nature of the care cascade for the condition touches on aspects of screening, linkage to various services, patient retention and the provision of long-term treatment. Any strategy that can effectively address viral hepatitis could be applicable to other communicable diseases associated with migration.

## Why target migrant subgroups?

The novel, affordable treatments provide a unique opportunity to eliminate viral hepatitis globally. Furthermore, the replacement of liver biopsies by non-invasive tests has enabled a major obstacle in the care cascade to be overcome. However, the impact of these developments has been tempered by the need for an effective public health strategy to ensure adequate disease prevention, monitoring and treatment coverage. Predominantly, tertiary care centres manage patients with HCV infection. These centres have strict treatment eligibility criteria, which may involve the diagnosis of cirrhosis, measurement of the HCV viral load, detection of the HCV genotype and the need to abstain from drugs and alcohol before accessing HCV therapy. However, there is no evidence for the necessity of drug and alcohol abstention, which has been a major obstacle to receiving care.^25^ In addition, the treatment of HBV infection is complex as patients must be continually assessed for eligibility, in contrast to HCV and the human immunodeficiency virus (HIV), where the recommendation is to treat all those infected. Consequently, patients with an HBV infection must attend multiple follow-up visits involving highly specialized assessments that are usually carried out only in tertiary care centres.^26^

The complexity of current models of care presents barriers to all migrant groups. In addition, particular subgroups, such as undocumented migrants and asylum seekers, face further obstacles: they may be excluded from the general health-care system because host nations have implemented hostile laws or because sociocultural factors deter them from accessing care. Despite these hurdles, there are several reasons why these subgroups must be targeted if viral hepatitis is to be eliminated in the European Economic Area or, indeed, globally.

First, the burden of viral hepatitis, especially of HBV-associated hepatitis, in the European Economic Area is driven by migrant movements from highly endemic nations. In 2016, it was estimated that 53% of 49 million migrants born outside of the European Economic Area came from nations where the endemicity of HBV infection was either intermediate (i.e. above 2%) or high (i.e. above 8%).^27^ Moreover, the prevalence of HBV infection among migrants to the EU who were born in highly endemic regions was 5% compared with 1% in the general population.^27^ The European Centre for Disease Prevention and Control estimated that the prevalence of HBV infection among documented migrants born in countries where the disease was endemic was 6%.^27^ The prevalence in refugees and asylum seekers has been reported to be even higher than in other migrants: 10% versus 5%, respectively.^28^ Furthermore, in Canada in 2013, the incidental antenatal rate for the diagnosis of HBV infection was six times higher in migrant women than in the general population.^29^ Similarly, among an estimated 4.2 million adults in the European Economic Area with a chronic HCV infection, the prevalence of anti-HCV antibodies was 2% in migrants from endemic countries compared with 1% in the general population.^8^

Second, chronic viral hepatitis follows a prolonged asymptomatic course during which infected individuals are unaware of their infection, until they reach an advanced disease stage. About 40–80% of people with chronic hepatitis virus infections worldwide are unaware of their infective status.^30^ This fact may make the healthy migrant concept less relevant for viral hepatitis because chronic carriers are often asymptomatic and can seemingly be in good health. In addition, certain migrants are less likely to be aware of their infective status either because there is little health provision in their native countries or because they form communities within host nations that are difficult to reach. Despite the availability of treatment, diagnostic rates for HBV and HCV infection are remarkably variable. In 2017, only an estimated 9% of the 257 million people with a chronic HBV infection and an estimated 20% of the 71 million with a chronic HCV infection were diagnosed globally.^1^^,^^3^^,^^31^ Rates were particularly low in the African Region, India and Pakistan, where 3% or less of people with an HBV infection received a diagnosis.^1^^,^^3^

Finally, challenges exist even after screening for viral hepatitis has been performed. Linking individuals to care and, thereafter, maintaining contact with them for long-term monitoring or therapy have presented difficulties in various settings worldwide. The development of simpler techniques for measuring liver fibrosis (e.g. transient elastography) has made the task easier.^3^ Nevertheless, transient elastography and many laboratory tests (e.g. for measuring viral load) are available only in tertiary care centres. Furthermore, because the need for treatment changes over time and the care cascade is centralized, patients must attend multiple follow-up visits at tertiary clinics for viral load and fibrosis measurement.^26^ Even after linkage to tertiary care has been achieved successfully, simple factors such as language may deter follow-up. For example, a Dutch study found that language insufficiency alone was a major barrier to the retention of HBV-infected patients in care.^32^

In summary, certain migrant subgroups are: (i) more at risk of viral hepatitis because of their previous exposure to specific risk factors; (ii) more at risk of their condition being undiagnosed than they would have been in their native country or compared with the general population in their host nation; and (iii) more at risk of failing to be retained within the health-care system because of complex social factors and growing hostility within the system. Consequently, these subgroups must be specifically targeted to ensure they receive effective treatment and, moreover, to enable viral hepatitis to be eliminated within Europe. In many ways, migrant communities in host nations exist in a milieu similar to that in their native countries. Reaching these communities may, therefore, depend on using robust, local measures that have been tried and tested before in similar settings internationally.

## Lessons from low-resource countries

Although the management of viral hepatitis varies enormously around the world, reaching marginalized migrant communities may be possible by adopting innovations that focus on decentralizing and expanding care and treatment. There are many ways in which this can be achieved. In low-resource settings, for example, where access to laboratory facilities can be difficult, guidelines on the management of infectious diseases have helped simplify care for HIV-infected patients by advocating innovative, low-technology, analytical techniques, such as dried blood spot testing to assess viral loads.^33^ In fact, dried blood spot testing has been found to be reliable for measuring the viral loads of both HBV and HCV.^34^^,^^35^

Task-shifting, that is, specialized jobs are performed by less-specialized workers, has been crucial for scaling up services and improving treatment coverage for other infectious diseases, such as tuberculosis and HIV infection. This approach counters the disadvantages (e.g. the geographical, cultural and financial obstacles) of clinical services being concentrated in tertiary care centres. Clearly, the need for task-shifting is greatest in low-resource regions where there are critical shortages of most health-care workers – some countries have 100 times fewer doctors per capita than EU nations. For HBV and HCV infections, the availability of effective treatments with few side-effects considerably increases the possibility of task-shifting. A meta-analysis showed that task-shifting increased uptake of HBV testing fourfold in migrant communities when it was performed by culturally appropriate health-care workers.^36^ Furthermore, expert opinion has tended to favour decentralizing the provision of HCV therapy by task-shifting to community-based models of care.^3^ For example, in Egypt, recent implementation of national, facility-based screening for HCV infection in symptomatic individuals resulted in the identification of thousands of infected individuals, who were then linked to care.^5^^,^^37^

Jointly with task-shifting, targeting specific communities and adopting a proactive approach to screening have helped identify migrants with viral hepatitis and subsequently link them to care.^3^^,^^38^ In 2018, a position statement by the European Association for the Study of the Liver on immigration and viral hepatitis recommended early targeted screening programmes for migrants, ideally at the port of arrival, to ensure quick access to treatment.^12^ In addition, some countries have adopted the approach of targeting specific groups within communities. For example, in the Netherlands, high linkage to care was achieved for Chinese migrants who were recruited and screened at specific locations, such as schools, community centres, churches and local public health clinics.^39^ Similarly, a study in the United Kingdom of Great Britain and Northern Ireland showed that a direct approach to hepatitis screening in primary care was more effective than distributing leaflets opportunistically in mosques.^40^ Also, several studies in emergency departments in London successfully employed an opt-out strategy for viral hepatitis screening motivated by the knowledge that the only health care some migrants seek may be via the emergency services.^41^^–^^43^

The transition to community-based diagnosis and monitoring requires tools that can be accessed by decentralized facilities, while ensuring they remain diagnostically valid. Simple laboratory scores have been developed and used in low-resource regions as alternative tools for assessing liver disease severity and treatment eligibility: they include (i) the aspartate aminotransferase-to-platelet ratio index (APRI); (ii) the FIB-4 score; and (iii) the TREAT-B score, which is based on hepatitis B virus e antigen serostatus and the alanine aminotransferase level.^44^ For patients with an HCV infection, the APRI and the FIB-4 score have high negative predictive values for cirrhosis (e.g. an APRI < 1 has a 93.0% negative predictive value) and are universally available.^45^ In contrast, the performance of the APRI and the FIB-4 score in HBV-infected patients in Africa has been reported to be poor (area under the receiver operating characteristics 0.70 and 0.73, respectively).^46^^,^^47^ However, the TREAT-B score has been validated in West Africa for identifying HBV-infected patients in need of treatment and has very good sensitivity and specificity (score of > 2 constitutes 85% sensitivity and 77% specificity).^44^ Furthermore, rapid diagnostic tests have simplified diagnosis, and point-of-care technologies are increasingly replacing traditional, laboratory-based, serological testing. In 2016, a systematic review of HCV core antigen testing for diagnosing HCV infection reported that the assays being developed had high sensitivity (93.4%) and specificity (98.8%).^48^ In addition, dried blood spot testing has been demonstrated to have adequate accuracy for diagnosing HBV infection (sensitivity 98% and specificity 100% for hepatitis B surface antigen) and has the further advantage that samples can be easily stored and transported and can be analysed at a later date.^49^

If these assessment tools and diagnostic tests can be applied in European countries to provide a decentralized screening and monitoring system for displaced and marginalized communities, including high-risk migrant subgroups, the uptake and continuity of care for these individuals could be dramatically increased. Once this step has been achieved, the emphasis should be on providing neglected communities with the normal standard of care, without prejudice or political bias. This implementation must be rigorously monitored.

## Conclusion

In this interconnected world, complex sociopolitical factors hinder the reach of migrant communities in Europe who require treatment for viral hepatitis. Moreover, development of strategies addressing the consequences of rapid global movements of people into the European Economic Area from regions where viral hepatitis is highly endemic is essential. Relative to the impact of the current COVID-19 pandemic, viral hepatitis ultimately forms a small part of the overall discourse on population health. Nevertheless, if international efforts and global policies can be directed towards addressing this substantial public health concern, the result may be better understanding of how to improve health care as a whole for marginalized groups in Europe. Overall, the changes required begin and end with our perception of, and our attitude towards, people in these groups. Health-care workers have a particular responsibility when dealing with the most vulnerable in society and could challenge laws that state otherwise.
